# Effect of Intelligence Mindsets on Math Achievement for Chinese Primary School Students: Math Self-Efficacy and Failure Beliefs as Mediators

**DOI:** 10.3389/fpsyg.2021.640349

**Published:** 2021-03-26

**Authors:** Aoxue Su, Shuya Wan, Wei He, Lianchun Dong

**Affiliations:** College of Science, Minzu University of China, Beijing, China

**Keywords:** math achievement, Chinese student, intelligence mindsets, failure beliefs, math self-efficacy

## Abstract

This study examined the relationship of intelligence mindsets to math achievement for primary school students in the Chinese educational context, as well as the mediating function of math self-efficacy and failure beliefs in this relationship. Participants included 466 fifth graders (231 boys and 235 girls) from two Chinese primary schools. Results indicated that boys had significantly higher mean levels of growth mindsets and math self-efficacy than girls, whereas boys had no statistically significant differences to girls on failure beliefs and math grade. Further, intelligence mindsets had a significant positive effect on math achievement, and failure beliefs and math self-efficacy played a full mediating role in the relationship between intelligence mindsets and math achievement. Moreover, intelligence mindsets affected math achievement through the chain mediating role of failure beliefs and math self-efficacy. These above findings contribute to advance our knowledge about the underlying mechanisms through which intelligence mindsets affect math achievement, which are of great significance to students' growth and current educational practice.

## Introduction

Mathematics is a very important tool subject, which occupies students' learning life from kindergarten to university and even higher level. Studies have established that a solid foundation of mathematics is essential to the professionalization of all professions. If you are not good at mathematics, it is difficult to engage in activities related to STEM (Science, Technology, Engineering, and Math), let alone engage in STEM careers (Wang and Degol, 2017).

However, the idea that math is only for some people has deep roots in the field of mathematics. Researchers surveyed scholars in various disciplines at US universities and found that among all STEM fields, math scholars were the most extreme in emphasizing fixed, innate abilities (Leslie et al., 2015). The single belief—that math is a “gift” that some people have and others do not—is responsible for much of the widespread math failure and underachievement in the word (Boaler and Dweck, 2016).

In fact, almost all students have the ability to learn math well and enjoy it, which depends on the individual's mindset. Everyone has a intelligence mindset (also called implicit theory of intelligence), a basic belief about whether intelligence is fixed or malleable (Dweck, 2006). Two different types of intelligence mindsets can be distinguished: growth mindset and fixed mindset. A growth mindset believes that intelligence or ability can be constantly developed and changed with people's experience and learning. Correspondingly, a fixed mindset, believes that intelligence is predetermined, limited and unchangeable. General beliefs about intelligence across domains have also been expended to the incremental or entity views of particular domains like STEM or stereotypically masculine tasks (Moè et al., 2009; Patterson et al., 2016).

Intelligence mindsets would be a major influence on individual's academic and emotional experience, leading to different cognition—emotion—behavior responses in the face of academic success, failure and challenge, which in turn affect an individual's learning behavior, academic achievement, learning motivation, and psychological health status. Research has indicated that individuals with different intelligence mindsets have differences in achievement goals, especially in their responses to failures. A fixed mindset orientation is more concerned about performance goals and focuses more on score, ranking, and grade. On the contrary, a growth mindset orientation values mastery goals and focuses more on the mastery of knowledge and the improvement of ability. In addition, in the face of difficult tasks, a growth mindset orientation with mastery goals shows more resilience and makes more efforts to analyze their “not yet acquired” abilities and methods to overcome the difficulties. However, a fixed mindset orientation with performance goals will shrink back when they encounter challenges and difficulties, and they will be more likely to believe that the difficulties are due to the limitation of their own abilities. Therefore, individuals with growth mindsets have stronger learning motivation and self-efficiency, become more actively involved in learning, and improve their grades faster (Dweck, 2006).

### Intelligence Mindsets and Academic Achievement

Students' intelligence mindsets have an essential role in their academic achievement. A review of findings based on the relevant articles published from 1998 to 2017 illustrated that intelligence mindsets served to affect academic achievement in most studies (Zhang et al., 2017). Similar results were found in the Programme for International Student Assessment (PISA). For example, data analysis results of PISA 2012 showed that, on average across OECD countries, the highest achieving mathematics students were those with a growth mindset, and they outranked their counterparts by the equivalent of more than a year of mathematics (Boaler and Dweck, 2016). Also in PISA 2018, students with a growth mindset scored 32 points higher in reading than those with a fixed mindset, after accounting for the socio-economic profile of students and schools (Schleicher, 2019).

However, the impact of intelligence mindsets on academic achievement is not stable and regional and cultural differences might exist. Students from Asia, Oceania, and North America were reported to have a positive correlation between growth mindset and academic achievement, while students from Europe showed a positive correlation between fixed mindset and academic achievement (Costa and Faria, 2018). Fixed-oriented individuals are eager to get good grades to prove their own ability. However, growth-oriented individuals do not attach great importance to achievements; vs. the belief that good achievements are a byproduct of their love for learning (Dweck, 2006). Therefore, the effect of intelligence mindsets on academic achievement may have complex psychological mechanisms, which have received only little attention.

### Academic Self-Efficacy and Failure Beliefs as Mediators

#### Academic Self-Efficacy as a Mediator

Academic self-efficacy refers to the belief in one's capabilities to master new skills and tasks in a specific academic domain such as mathematics (Bandura, 1997). Previous studies have reached a consensus that academic self-efficacy was an important construct to explain students' achievement-related behaviors related to learning and performance (Schunk, 1989; Pajares, 1996; Chemers et al., 2001; Choi, 2005; Komarraju and Nadler, 2013; Macphee et al., 2013). Self-efficacy beliefs can not only predict a student's performance in mathematics such as the accuracy of mathematical operations and the ability of mathematic problem-solving (Schunk and Hanson, 1985; Pajares and Miller, 1994), but also can decrease mathematics anxiety (Samuel and Warner, 2019). Also, it has demonstrated that students with a stronger self-efficacy showed greater persistence on difficult math items than those with lower self-efficacy (Collins, 1982).

Students' intelligence mindsets may play a role through the stable academic self-efficacy within individuals. Martocchio (1994) found that self-efficacy increased for students with a growth mindset vs. decreased for those with a fixed mindset in the face of a challenging computers course. Samuel and Warner (2019) found that college students' self-efficacy in math was increased through a combination intervention of mindfulness and intelligence mindsets. Mcwilliams (2014) found that students with a growth mindset tend to make internal attributions and have a strong sense of academic self-efficacy. Additionally, results of PISA 2018 also indicated that a growth mindset was positively correlated with students' general self-efficacy.

However, students from grade 6 to 8 who received special education due to reading disabilities were investigated, and an intelligence mindsets intervention was conducted on the experimental group. Results showed that this intervention could significantly improve the learning motivation level of the experimental group, but there was no significant difference in self-efficacy and academic achievement between the experimental group and control group (Rhew et al., 2018).

The inconsistent results of previous studies may be due to the fact that the role of academic self-efficacy has not been fully explored and needs to be further investigated.

#### Failure Beliefs as a Mediator

Failure beliefs (Nishimura et al., 2017; Stern and Hertel, 2020) are a way of thinking that views failures as either an enhancing or debilitating experience. Different failure beliefs would lead to different characteristic response patterns to academic difficulties. A failure-is-enhancing belief views failure as an enhancing experience that promotes learning. In the face of academic failures, individuals with this kind belief are more likely to adopt effort-based attributions and then would to engage in positive, effort-based coping strategies. In contrast, a failure-is debilitating belief views failure as an impairing experience that inhibits learning. Those students tend to adopt ability-based attributions and then would engage in negative, effort-avoidant coping strategies when encountering academic difficulties (Haimovitz and Dweck, 2016).

Different failure beliefs could lead to different learning outcomes. Dweck and Gilliard (1975) found that altering attributions for failures from low-ability to low-effort would enable learned helpless children to improve their problem-solving ability. Blackwell et al. (2007) established that students who make fewer ability-based helpless attributions would choose more positive, effort-based strategies to copy with failures, improving their math scores.

Different intelligence mindsets would set up different patterns of response to the threat of failures (Dweck et al., 1995b; Robins and Pals, 2002; Whittington et al., 2017). Relative to those with a fixed mindset, students with a growth mindset have been found to express less fear of failure and set up a more mastery-oriented pattern rather than a helpless response pattern in the face of academic setbacks. Specifically, they were more likely to make low-effort instead of low-ability attributions for failures and apt to employ positive strategies, such as the development of better strategies and work harder under failure, rather than negative strategies, such as an avoidance of challenge and effort withdrawal.

Students' intelligence mindsets may have few effects on academic achievement until challenges or setbacks or failures are present (Dweck, 2002, 2008; Grant and Dweck, 2003). In other words, the effect of intelligence mindsets on achievement becomes stronger in the face of failure. Therefore, the effect of intelligence mindsets on math achievement in challenging and demanding situations should be further examined.

#### Academic Self-Efficacy and Failure Beliefs as a Chain Mediator

Academic self-efficacy and failure beliefs were associated with one another. Effort attribution feedback on success or failure can increase students' academic self-efficacy (Schunk, 1989). Conversely, the more individuals attribute failure to ability and task difficulties, the lower their expectations of future success (Weiner, 1986). Attribution style was also found as the strongest predictor of self-confidence in math (Kloosterman, 1988). Moreover, the ways educators discuss success, failure, and challenges with students can also have a strong impact on improving academic self-efficacy. Educators can help students build self-efficacy by portraying failure as a positive aspect of learning while emphasizing the importance of persisting in overcoming these challenges (Rhew, 2017).

At the same time, academic self-efficacy has been revealed to play important roles in shaping people's attributions for failures and in their behavioral responses to attributions for failures (Dixon and Schertzer, 2013). Students with low self-efficacy may avoid accomplishing a task, whereas those who believe in their abilities should participate more eagerly. Especially in the face of setbacks and failures, a confident person ought to work harder and persist longer than those who doubt their abilities (Schunk, 1989). Similarly, in the face of failures, students with high self-efficacy would make low-effort attributions, while those with low self-efficacy would make low-ability attributions (Ganguly et al., 2017; Song et al., 2020).

### Influence of Gender

For a long time, traditional math-gender stereotypes were very popular. Mathematics was considered as a “male subject,” that is, males are good at math and perform better in math than females (Cvencek et al., 2011; Moè, 2018). In contrast, traditional math-gender stereotypes were rejected in some studies, where girls are believed to be as good as boys in math or even perform better in math than boys (Passolunghi et al., 2014). Based on the analysis of empirical data, it is also found that contradictory results often occur in the gender difference in math achievement. For example, in PISA 2012, out of 72 participating countries (regions or economies), boys' math scores were statistically significantly higher than girls' in 28 countries (regions or economies), while girls' math scores were significantly higher than boys' in 7 countries.

Math-gender stereotypes were found to affect both boys' and girls' self-perception of math ability. Therefore, the gender difference in math self-efficacy is also inconsistent. Several findings have indicated that girls had lower levels of math self-efficacy than boys (Middleton, 1999; Diseth et al., 2014). In contrast, other studies have observed the opposite, that is, girls were more self-efficacious in math than boys (Guvercin, 2008). Also, the well-established gender difference in math self-efficacy was not observed in some studies, that is, gender had no significant effect on math self-efficacy (Passolunghi et al., 2014).

Regarding gender differences in intelligence mindsets, few studies have been conducted. Findings obtained by Spinath et al. (2003) suggested a significant positive correlation with growth mindset for women. While Diseth et al. (2014) found that girls had weaker growth mindsets than boys. Gender was also found to be unrelated to intelligence mindset in other studies (Burnette, 2013).

Concerning gender differences in failure beliefs, compared to boys, girls (especially high-achieving girls) were reported to have a lower tendency for new and challenging tasks and tend to endorse ability-based attributions (Chen, 2012). Whereas some studies reported that very small differences in failure or success attribution exist among boys and girls no matter they are advantaged or disadvantaged SES (Bar-Tal et al., 1984).

Overall, previous studies represent high inconsistency and more studies are needed to illuminate the influence of gender.

### The Present Study

These previous findings summarized above show that intelligence mindsets, failure beliefs, math self-efficacy and math achievement do correlate with each other, and it is very important and meaningful to understand the influencing mechanism between these variables. However, based on these prior findings, there are still some questions that need to be further investigated. First, the inconsistent results of previous studies as mentioned above call for further investigation of the relationship between these variables. These inconsistent results may be related to cultural background. For example, the theory of intelligence mindsets has been found to be culturally shaped in previous studies (Stevenson et al., 1990; Morris and Peng, 1994; Dweck et al., 1995b; Costa and Faria, 2018). However, at present, few researches have been conduct with Eastern cultures, such as Chinese culture (Zeng et al., 2016; Zhao et al., 2018). As far as we know, no empirical research has been conducted to exam the effect of intelligence mindsets on academic achievement in the context of Chinese education. Therefore, this study investigated, for the first time, the relationship of intelligence mindsets to math achievement for Chinese students.

Second, why is intelligence mindset related to math achievement? Although many researches have indicated that mindsets play important roles in math achievement, few studies have investigated the underlying mechanisms through which mindsets correlate with achievement (Blackwell et al., 2007). As far as we know, our full model of the relationships between intelligence mindsets, failure beliefs, math self-efficacy, and math achievement has never been investigated before. Therefore, this study would contribute to advance our knowledge about the underlying mechanisms through which intelligence mindsets affect math achievement.

Based on previous research findings and our theoretical model, the following hypotheses are proposed:

- Hypothesis 1: boys have higher levels of growth mindset, math self-efficacy, failure beliefs and math achievement than girls;
- Hypothesis 2: growth mindset is positively related to math achievement;
- Hypothesis 3: growth mindset can positively predict math self-efficacy, and math self-efficacy can positively predict math achievement, as well as playing a mediation role between intelligence mindsets and math achievement;
- Hypothesis 4: growth mindset can positively predict failure beliefs, and failure beliefs can positively predict math achievement, as well as playing a mediation role between intelligence mindsets and math achievement.
- Hypothesis 5: math self-efficacy can positively predict failure beliefs, as well as math self-efficacy and failure beliefs sequentially mediate the relationship between intelligence mindsets and math achievement.

## Materials and Methods

### Participants

We gathered convenient samples from two public primary schools in Urumqi, the capital city of Xinjiang Uygur Autonomous Region located in the northwest border of China. These two participating schools are located in the urban areas of Urumqi city, with various educational indicators that near the average education level of China. All fifth grade classes in each participated school, a total of eight classes, participated in this study.

Four hundred and sixty six fifth graders (ages ranging from 10 to 12 years) were recruited in total, which consisted of 231 (49.6%) boys and 235 (50.4%) girls. Participants were varied in ethnicity, among which 355 (76.2%) were Han, 45 (9.7%) were Uighurs, 43 (9.2%) were Hui, 10 (2.1%) were Kazak, and 13 (2.8%) were other nationalities.

A questionnaire survey was carried out in the classroom, taking a class as a unit, and within 15 min. One of the research assistants informed all participants that all of their responses would only be used for research purposes and encouraged them to provide honest answers in the questionnaire.

### Measures

All scale items were rated on a six-point Likert scale from 1 (*strongly agree*) to 6 (*strongly disagree*). Items were reverse-scored if necessary.

#### Intelligence Mindsets Scale

Three items (Dweck, 2006) were adopted to measure participants' fixed mindset, e.g., “you can't really change how intelligent you are.” Fixed mindset items rather than growth mindset items were chosen because growth items sometimes create an acquiescence bias (Claro et al., 2016). As two items in the scale were tautology after being translated into Chinese, only two items were retained in the final survey. These items were reverse-scored and then mean score of these two items was calculated as intelligence mindsets score, with a higher score indicating a stronger growth mindset (*M* = 4.53, SD = 1.35). The Cronbach's α coefficient for intelligence mindsets scale was 0.81.

#### Math Self-Efficacy Scale

Three items were selected from the PALS (Midgley et al., 2000) to measure participants' confidence in their ability to master math skills, e.g., “I am good at math.” The mean score of these three items was calculated as the math self-efficacy score, with a higher score indicating a higher confidence in their math ability (*M* = 5.14, SD = 0.75). The Cronbach's α coefficient for math self-efficacy scale was 0.73.

#### Failure Beliefs Scale

The failure beliefs scale consisted of failure attributions and coping strategies subscales. Four items were used to measure participants' characteristic response patterns to mathematical difficulties (Blackwell et al., 2007). Among these items, two were used to measure students' failure attributions, that is, students rated the extent to which they believed their abilities or other factors contributed to the failure, e.g., “if I failed to pass my math test, it's because I'm not smart enough.” The remaining two items were used to measure students' coping strategies for failures, that is, students rated how likely they were to adopt positive strategies, e.g., “if I failed to pass my math test, I would spend more time studying before the exam.” Some of the items were negative statements and therefore were reverse-scored before data analysis. Then mean score of these four items was calculated as failure beliefs score, with a higher score indicating a more positive response to failures (*M* = 5.37, SD = 0.7). The Cronbach's α coefficient for failure beliefs scale was 0.69.

#### Math Achievement

For many students, mathematics is a challenging subject that can trigger the distinctive motivational patterns associated with intelligence mindsets, which may not manifest themselves in low-challenge situations (Blackwell et al., 2007). Thus, math scores on the Urumqi's assessment of education quality in the spring term of fifth grade served as the measure of math achievement (*M* = 89.58, SD = 17.09, range = 0–100). Test questions of this assessment were mainly those that reflected the basic requirements of National Mathematics Curriculum for primary students. All fifth graders in Urumqi studied under the same mathematics curriculum and took the same exam.

### Data Analytic Procedures

Data analysis subsequently included the following steps. First, analysis of statistical description and correlations of all study measures were calculated with SPSS21.0 software. Second, independent sample *t*-tests were performed to test the mean differences between boys and girls regarding all the study measures. Third, structural equation modeling (SEM) was conducted to examine the relationships between all study measures using M-plus7.0 software. Fourth, a bootstrapping method was used to test the mediating effect of math self-efficacy and failure beliefs. Lastly, multi-group analysis was conducted to test the structural differences of the full model by genders.

## Results

### Common Method Bias Test

Common method biases may happen due to self-report methods, so Harman's single factor analysis was carried out to test the common method biases. Results showed that a total of four factors were extracted and the first factor explained 31.76% of the variance variation, which was less than the critical standard 40%, indicating that common method bias in this study was not obvious.

### Descriptive Analysis and Intercorrelations

Students were classified into three different mindset categories according to their average intelligence mindsets score (Claro et al., 2016): students who scored from 1 to 2 points were categorized as “fixed mindsets;” those who scored from 5 to 6 points were categorized as “growth mindsets;” and those who scored from 2.1 to 4.9 points were categorized as “mixed mindsets,” with 9.0, 58.2, and 32.8% falling into each category, respectively. Apparently, those participants were, more likely to have a growth mindset, which is consistent with previous research results under the same cultural background (Stevenson et al., 1990; Dweck et al., 1995a).

The distribution of math grades showed that 94% of the students scored above 60 and reached the basic requirements of the curriculum standard, which was in line with the results of national mathematics large-scale assessments for compulsory education (Liu et al., 2014). Chinese students have been outstanding in mastering basic math knowledge and basic skills for a long time and have excelled in international assessments of mathematics achievement (Ni et al., 2011). The present assessment, which focused on students' mastery of basic knowledge and basic skills, got an average score of 89.58 as expected. At the same time, the standard deviation was 17.09 and the minimum score was as low as 20, indicating a tendency toward polarization on math grade.

Correlation analysis results (see Table 1) showed that intelligence mindsets, math self-efficacy, failure beliefs, and math grade formed a network of interrelated variables as expected. Specifically, intelligence mindsets was significantly positively correlated with math self-efficacy (*r* = 0.126, *p* < 0.01), failure beliefs (*r* = 0.214, *p* < 0.01), and math grade (*r* = 0.166, *p* < 0.01). Math self-efficacy was significantly correlated with failure beliefs (*r* = 0.443, *p* < 0.01). Moreover, both math self-efficacy and failure beliefs were positively related to math grade (*r* = 0.319, *p* < 0.01; *r* = 0.301, *p* < 0.01).

**Table 1:** Descriptive analysis and intercorrelations.

**Measures**	**Range**	***M* ± *SD***	**1**	**2**	**3**	**4**
1. Intelligence mindsets	1–6	4.53 ± 1.35	-			
2. Math self-efficacy	1–6	5.14 ± 0.75	0.126^**^	-		
3. Failure beliefs	1–6	5.37 ± 0.70	0.214^**^	0.443^**^	-	
4. Math grade	0–100	89.58 ± 17.09	0.166^**^	0.319^**^	0.301^**^	-

N = 466,

** *p < 0.01*.

### Mean Differences

Independent sample *t*-tests were conducted to examine mean level differences of these variables regarding gender. As shown in Table 2, on average, boys had higher mean level scores on all the variables. However, statistically significant differences were found only on two variables. Compared to girls, boys have significantly higher mean levels of growth mindsets and math self-efficacy (*p* < 0.05). While no statistically significant differences were found between boys and girls on failure beliefs (*p* = 0.165) and math grade (*p* = 0.258).

**Table 2:** Mean differences by gender and *t*-test results.

**Measures**	**Range**	**Boys *M* ± *SD***	**Girls *M* ± *SD***	***t***
Intelligence mindsets	1–6	4.68 ± 1.32	4.37 ± 1.36	2.49^*^
Math self-efficacy	1–6	5.21 ± 0.78	5.07 ± 0.73	2.05^*^
Failure beliefs	1–6	5.42 ± 0.71	5.33 ± 0.69	1.39
Math grade	0–100	90.49 ± 17.60	88.69 ± 16.55	1.13

N = 231 for boys, N = 235 for girls,

* *p < 0.05*.

### Structural Equation Modeling

Structural equation modeling (SEM) was used to further examine the relationship between all study measures. First, measurement models were examined. Intelligence mindsets were indexed by two items; math self-efficacy was indexed by three items; failure beliefs were indexed by four items. Math grade was used as the outcome variable. All the factor loadings ranged from 0.456 to 0.842 and were significant, indicating that all the measurement indicators could be well-explained by the latent variables. Next, a structural model was conducted to establish the structural relationship between latent variables.

**Figure 1:**
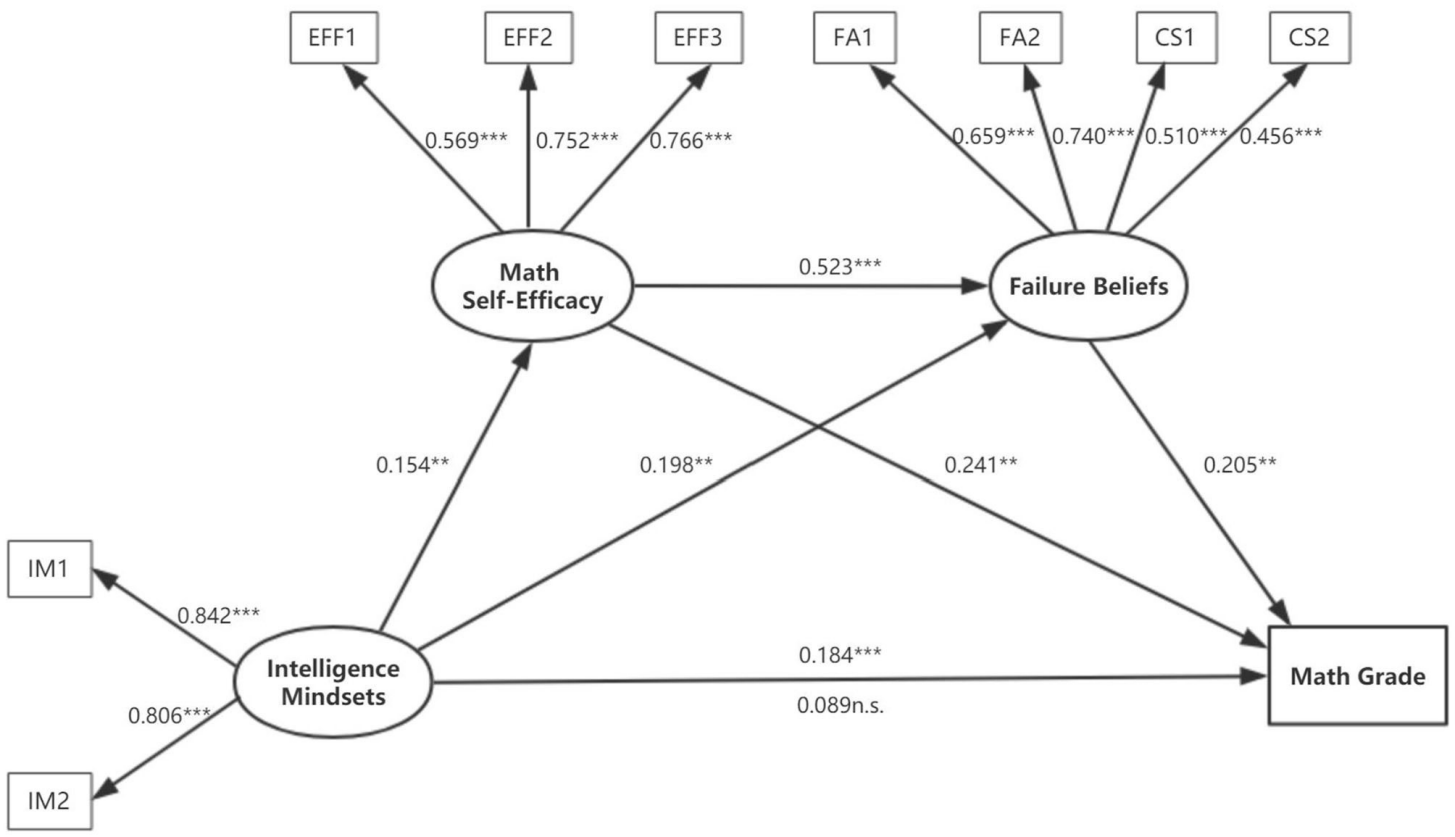
Full model. Along the bottom path, the coefficient above the arrow indicates the direct effect, and the coefficient below the arrow indicates the effect with mediators. **p < 0.01, ***p < 0.001.

Results showed that the full model (see Figure 1) was well-supported by the data (*CFI* = 0.943 > 0.9, *TLI* = 0.915 > 0.9, *SRMR* = 0.049 < 0.08, *RMSEA* = 0.067 < 0.08) (Hu and Bentler, 1999), and all proposed paths were significant. Growth mindset can directly predict students' math achievement, as well as indirectly predict students' math achievement through math self-efficacy and failure beliefs. To be specific, growth mindset significantly predicted math achievement (β = 0.184, *p* < 0.001), math self-efficacy (β = 0.154, *p* < 0.01), and failure beliefs (β = 0.198, *p* < 0.01); math self-efficacy significantly predicted math achievement (β = 0.241, *p* < 0.01) and failure beliefs (β = 0.523, *p* < 0.001); and failure beliefs significantly predicted math achievement (β = 0.205, *p* < 0.01).

### Mediating Modeling Analyses

The full model suggests that (a) failure beliefs mediate the relationship between intelligence mindsets and math grade, (b) math self-efficacy mediates the relationship between intelligence mindsets and math grade, and (c) failure beliefs and math self-efficacy chain mediate the relationship between intelligence mindsets and math grade. To further test whether the mediating effect was significant, a bootstrapping procedure with 10,000 bootstrap samples was used. If the bias-corrected (BC) 95% confidence interval (CI) for the path coefficient does not include 0, the mediating effect is significant.

As shown in Table 3, the direct relationship of intelligence mindsets and math grade (β = 0.184, *p* < 0.001) was found to be mediated by math self-efficacy [β = 0.037, *p* < 0.01, 95% *CI* = (0.007, 0.089)], failure beliefs [β = 0.041, *p* < 0.01, 95% *CI* = (0.005, 0.108)], and math self-efficacy to failure beliefs [β = 0.017, *p* < 0.01, 95% *CI* = (0.002, 0.054)]. The fact that the direct effect of intelligence mindsets on math grades was no longer significant after the model controlled for math self-efficacy and failure beliefs (β = 0.089, *p* > 0.05), which indicated a full mediation. The students with a growth mindset predicted a higher sense of math self-efficacy, and then predicted a more positive failure belief, which in turn contributed to students' math achievement.

**Table 3:** Bootstrapping analysis of the mediating effect.

**95%BC** ***CI***				
**Mediator**	**Parameter estimate**	**SE**	**Lower**	**Upper**
Math self-efficacy	0.037	0.021	0.007	0.089
Failure beliefs	0.041	0.025	0.005	0.108
Math self-efficacy → Failure beliefs	0.017	0.013	0.002	0.054

### Multigroup Analysis of the Full Model

In order to explore whether the full model depicted in Figure 1 is equally valid across genders, a multi-group analysis was conducted (Vandenberg and Lance, 2000; Yao and Yang, 2017). As shown in Table 4, Model 1 (unconstrained model) has the restriction that all coefficients allowed to vary across genders; Model 2 restricted the measurement weights to be equal; Model 3 restricted the measurement weights and structural weights to be equal; In Model 4 (constrained model), all coefficients, including measurement weights, structural weights, structural covariances, structural residuals, and measurement residuals, were set invariant across genders. The χ^2^ differences among these four models were not significant (all *p*s > 0.05), indicating that the structural relationships shown in Figure 1 were not found to have a significant difference for boys and girls. The generalizability of the full model was preliminarily supported.

**Table 4:** Results of multi-group analysis: boys vs. girls.

**Model**	**Specifications**	**χ^2^**	***df***	**CFI**	**RMSEA**	**Model comparison**	**χ^2^ diff**.	**df diff**.	***p***
1	Unconstrained	134.018	60	0.934	0.052				
2	Measurement weights equal	146.117	69	0.931	0.049	1 vs.2	12.099	9	0.208
3	Structural weights equal	149.403	72	0.931	0.048	2 vs.3	3.286	3	0.350
4	Constrained	170.153	85	0.924	0.046	3 vs.4	20.75	13	0.078

## Discussion

This study aimed to explore the influencing mechanism of intelligence mindsets on math achievement for Chinese primary school students. SEM suggested that intelligence mindsets, math self-efficacy, and failure beliefs could all predict math achievement. Moreover, mediating modeling analyses further suggested that the association of intelligence mindsets and math achievement could be fully explained by math self-efficacy and failure beliefs. So we conclude that math self-efficacy and failure beliefs are meaningful concepts for understanding the mechanism of intelligence mindsets on math achievement. Specifically, the results revealed that having a growth mindset predicted a higher sense of math self-efficacy as well as more positive failure beliefs, and also having a higher sense of math self-efficacy predicted more positive failure beliefs, which both in turn positively influenced students' math achievement. Regarding the gender difference, our findings showed that boys had significantly higher mean levels of growth mindsets and math self-efficacy than girls, while boys and girls had no statistically significant differences on failure beliefs and math grade. In addition, the full model was proved to be equally valid across genders and the generalizability of the full model was preliminarily supported by the multi-group analysis. In summary, hypotheses 2, 3, 4, and 5 are all supported while hypothesis 1 is partially confirmed.

In the previous literatures, several paths have been examined separately. Our findings are in line with previous studies on the following: (a) intelligence mindset, math self-efficacy and failure beliefs could contribute to one's math achievement (Dweck and Gilliard, 1975; Dweck and Leggett, 1988; Schunk, 1989; Chemers et al., 2001; Blackwell et al., 2007; Boaler and Dweck, 2016; Claro et al., 2016); (b) growth mindset predicts math self-efficacy (Martocchio, 1994; Samuel and Warner, 2019); (c) growth mindset is positively related with failure beliefs (Dweck et al., 1995b; Robins and Pals, 2002; Whittington et al., 2017); and (d) math self-efficacy and failure beliefs are positively correlated (Schunk, 1989; Dixon and Schertzer, 2013; Ganguly et al., 2017). However, as far as we know, the full paths of the relationships among intelligence mindset, math self-efficacy, failure beliefs, and math achievement in our mediating model have not been tested simultaneously before in other research.

Most importantly, this study highlights the critical mediating roles of failure beliefs in the relationship between intelligence mindsets and math achievement. That is, intelligence mindsets can play a more important role in students' math achievement when faced with challenges, setbacks, or failures. For students with a growth mindset, a failure indicates that more effort needs to be put into the task in order to improve their intelligence or basic ability to do the task well, so they are more likely to attribute the failure to insufficient effort. In turn, these students with a belief of positive effort will tend to adopt positive strategies, such as persistence on the tasks and invest efforts to solve these problems in the face of challenges, setbacks, and failures, thereby improving math grades. By comparison, for students with a fixed mindset, a failure represents low intelligence or ability that cannot be developed through effort and hard work, so they are more likely to attribute their failure to their ability. In turn, those students with ability beliefs apt to employ negative strategies, such as an avoidance of study challenges and effort withdrawal in face of setbacks, which leaded to flat or even falling math grades over time.

Likewise, this study also highlights the critical mediating roles of math self-efficacy as well as the chain mediating roles of math self-efficacy and failure beliefs in the relationship between intelligence mindsets and math achievement. Students holding a growth mindset or fixed mindset have very different perspectives in views of math self-efficacy. Students with a growth mindset believe that their intelligence and ability can be improved over time. Thus, they have a higher belief in their own capabilities and participate more eagerly for accomplishing a task than students who thought their intelligence and ability was fixed. Especially when facing failures, individuals who feel efficacious ought to make effort attributions and then work hard. Therefore, they were outperforming those who held more fixed mindsets and following low self-efficacy in mathematics.

A series of studies have proved that intelligence mindsets can be cultivated and a fixed mindset can also be transformed into a growth mindset by interventions (Blackwell et al., 2007). For example, students can be taught about the new science of brain plasticity and the new view of talent and giftedness as dynamic attributes that can be developed. At the same time, students can be guided to focus on effort and process through process praise and feedback by parents and teachers. Especially for the females and minority students, such messages should be conveyed that their underachievement has its roots in environmental rather than intelligence factors, and can be overcome through the improvement of the education environment and individual efforts (Blackwell et al., 2007; Dweck, 2008). Most importantly, our findings further demonstrated that in order to play a stable role on math achievement, intelligence mindsets need to be applied with the help of positive academic self-efficacy and failure beliefs. Therefore, parents and teachers should train students to develop self-motivated and self-directed growth orientations, give positive feedback to students when they face challenges and setbacks, and encourage them to meet challenges, persist, and become more confident. At the same time, parents and teachers should guide students to establish a correct view of mistakes, let them know the value of failures, realize that making mistakes is the best time to learn and a key time for brain growth, then let students learn from mistakes, and thus achieve the goal of improving academic achievement.

### Limitations

This present research has demonstrated that math self-efficacy and failure beliefs are meaningful concepts for understanding the mechanism of intelligence mindsets on math achievement for the first time. However, this study also has some limitations. First, the study was conducted in two schools in Urumqi. It provided a window for relevant researchers to understand the underlying mechanisms through which intelligence mindsets are related to math achievement in the context of Chinese education. Although the educational indicators of these two schools were close to Chinese average education level, the samples were not gathered based on the probability sampling method, which may raise the question of generalization. Further studies should be conducted in larger samples to assess whether the findings of this study are still valid. Second, cross-sectional data was collected in this study, which is insufficient to understand how the positive role of intelligence mindsets is played vertically. Longitudinal approaches should be conducted in future studies to examine the vertically positive role. Finally, this study only used self-reports of primary school students, and future studies should combine perspectives of parents and teachers to further explore the influence of external environment on individual's intelligence mindsets.

## Data Availability Statement

The raw data supporting the conclusions of this article will be made available by the authors, without undue reservation.

## Ethics Statement

The studies involving human participants were reviewed and approved by Ethics Review Committee at College of Scinece, Minzu University of China. Written informed consent to participate in this study was provided by the participants' legal guardian/next of kin.

## Author Contributions

AS: research design, data collection, manuscript draft, and revision work. SW: literature search and data interpretation. WH: data collection and revision work. LD: data collection. All authors contributed to the article and approved the submitted version.

## Conflict of Interest

The authors declare that the research was conducted in the absence of any commercial or financial relationships that could be construed as a potential conflict of interest.

## References

[B1] Bandura A. (1997). Self-Efficacy: The Exercise of Control. New York, NY: Freeman.

[B2] Bar-Tal D. Goldberg M. Knaani A. (1984). Causes of success and failure and their dimensions as a function of ses and gender:a phenomenological analysis. Br. J. Educ. Psychol. 54, 51–61. 10.1111/j.2044-8279.1984.tb00844.x

[B3] Blackwell L. S. Trzesniewski K. H. Dweck C. S. (2007). Implicit theories of intelligence predict achievement across an adolescent transition: a longitudinal study and an intervention. Child Dev. 78, 246–263. 10.1111/j.1467-8624.2007.00995.x 17328703

[B4] Boaler J. Dweck C. S. (2016). Mathematical Mindsets: Unleashing Students' Potential Through Creative Math, Inspiring Messages, and Innovative Teaching. Chappaqua, NY: John Wiley and Sons.

[B5] Burnette J. L. (2013). Mind-sets matter: a meta-analytic review of implicit theories and self-regulation. Psychol. Bull. 139, 655–701. 10.1037/a0029531 22866678

[B6] Chemers M. M. Hu L. Garcia B. F. (2001). Academic self-efficacy and first year college student performance and adjustment. J. Educ. Psychol. 93, 55–64. 10.1037/0022-0663.93.1.55

[B7] Chen J. A. (2012). Implicit theories, epistemic beliefs, and science motivation: a person-centered approach. Learn. Individ. Dif. 22, 724–735. 10.1016/j.lindif.2012.07.013

[B8] Choi N. (2005). Self-efficacy and self-concept as predictors of college students' academic performance. Psychol. Sch. 42, 197–205. 10.1002/pits.20048

[B9] Claro S. Paunesku D. Dweck C. S. (2016). Growth mindset tempers the effects of poverty on academic achievement. Proc. Natl. Acad. Sci. U.S.A. 113:8664. 10.1073/pnas.1608207113 27432947 PMC4978255

[B10] Collins J. (1982). Self-efficacy and ability in achievement behavior. Dissertation Abstracts International 46.

[B11] Costa A. Faria L. (2018). Implicit theories of intelligence and academic achievement: a meta-analytic review. Front. Psychol. 9:829. 10.3389/fpsyg.2018.00829 29922195 PMC5996155

[B12] Cvencek D. Meltzoff A. N. Greenwald A. G. (2011). Math–gender stereotypes in elementary school children. Child Dev. 82, 766–779. 10.1111/j.1467-8624.2010.01529.x 21410915

[B13] Diseth A. Meland E. Breidablik H. J. (2014). Self-beliefs among students: grade level and gender differences in self-esteem, self-efficacy and implicit theories of intelligence. Learn. Individ. Dif. 35, 1–8. 10.1016/j.lindif.2014.06.003

[B14] Dixon A. L. Schertzer S. M. B. (2013). Bouncing back: how salesperson optimism and self-efficacy influence attributions and behaviors following failure. J. Pers. Selling Sales Manage. 25, 361–369. 10.1080/08853134.2005.10749070

[B15] Dweck C. S. (2002). The development of ability conceptions, in Development of Achievement Motivation (Elsevier), 57–88. 10.1016/B978-012750053-9/50005-X

[B16] Dweck C. S. (2006). Mindset: The New Psychology of Success. New York, NY: Random House Incorporated.

[B17] Dweck C. S. (2008). Mindsets and Math/Science Achievement.

[B18] Dweck C. S. Chiu C.-y. Hong Y.-y. (1995a). Implicit theories and their role in judgments and reactions: a world from two perspectives. Psychol. Inq. 6, 267–285. 10.1207/s15327965pli0604_1

[B19] Dweck C. S. Chiu C.-y. Hong Y.-y. (1995b). Implicit theories: elaboration and extension of the model. Psychol. Inq. 6, 322–333. 10.1207/s15327965pli0604_12

[B20] Dweck C. S. Gilliard D. (1975). Expectancy statements as determinants of reactions to failure: sex differences in persistence and expectancy change. J. Pers. Soc. Psychol. 32, 1077–1084. 10.1037/0022-3514.32.6.1077

[B21] Dweck C. S. Leggett E. L. (1988). A social-cognitive approach to motivation and personality. Psychol. Rev. 95, 256–273. 10.1037/0033-295X.95.2.256

[B22] Ganguly S. Kulkarni M. Gupta M. (2017). Predictors of academic performance among Indian students. Soc. Psychol. Educ. 20, 139–157. 10.1007/s11218-016-9345-y

[B23] Grant H. Dweck C. S. (2003). Clarifying achievement goals and their impact. J. Pers. Soc. Psychol. 85, 541–553. 10.1037/0022-3514.85.3.541 14498789

[B24] Guvercin Ö. (2008). Investigating Elementary Students' Motivation Towards Science Learning: A Cross Age Study. Ankara: Middle East Technical University.

[B25] Haimovitz K. Dweck C. S. (2016). What predicts childrens fixed and growth intelligence mind-sets? Not their parents views of intelligence but their parents views of failure. Psychol. Sci. 27, 859–869. 10.1177/0956797616639727 27113733

[B26] Hu L. Bentler P. M. (1999). Cutoff criteria for fit indexes in covariance structure analysis: conventional criteria versus new alternatives. Struct. Equation Model. 6, 1–55. 10.1080/10705519909540118

[B27] Kloosterman P. (1988). Self-confidence and motivation in mathematics. J. Educ. Psychol. 80, 345–351. 10.1037/0022-0663.80.3.345

[B28] Komarraju M. Nadler D. (2013). Self-efficacy and academic achievement: why do implicit beliefs, goals, and effort regulation matter? Learn. Individ. Dif. 25, 67–72. 10.1016/j.lindif.2013.01.005

[B29] Leslie S. J. Cimpian A. Meyer M. Freeland E. (2015). Expectations of brilliance underlie gender distributions across academic disciplines. Science 347, 262–265. 10.1126/science.aaa9892 25593183

[B30] Liu J. Zhang D. Qi C. Cao Y. (2014). Study on status and influence factors of mathematics academic achievements of compulsory education in Mainland China. Glob. Educ. 43, 44–57.

[B31] Macphee D. Farro S. Canetto S. S. (2013). Academic self-efficacy and performance of underrepresented STEM majors: gender, ethnic, and social class patterns. Anal. Soc. Issues Publ. Policy 13, 347–369. 10.1111/asap.12033

[B32] Martocchio J. J. (1994). Effects of conceptions of ability on anxiety, self-efficacy, and learning in training. J. Appl. Psychol. 79:819. 10.1037/0021-9010.79.6.819 7852207

[B33] Mcwilliams E. C. (2014). Self-efficacy, implicit theory of intelligence, goal orientation and the ninth grade experience (Dissertations and Theses). Northeastern University, Boston, MA, United States.

[B34] Middleton M. J. (1999). Gender differences in academic self-effcacy: the role of promoting classroom interaction, in Paper Presented at the American Educational Research Association (Montreal, QC).

[B35] Midgley C. Maehr M. L. Hruda L. Z. Anderman E. Anderman L. Freeman K. E.. (2000). Manual for the Patterns of Adaptive Learning Scales. Ann Arbor, MI: University of Michigan.

[B36] Moè A. (2018). Mental rotation and mathematics: gender-stereotyped beliefs and relationships in primary school children. Learn. Individ. Dif. 61, 172–180. 10.1016/j.lindif.2017.12.002

[B37] Moè A. Meneghetti C. Cadinu M. (2009). Women and mental rotation: incremental theory and spatial strategy use enhance performance. Pers. Individ. Dif. 46, 187–191. 10.1016/j.paid.2008.09.030

[B38] Morris M. W. Peng K. (1994). Culture and cause: American and Chinese attributions for social and physical events. J. Pers. Soc. Psychol. 67, 949–971. 10.1037/0022-3514.67.6.949

[B39] Ni Y. Li Q. Li X. Zhang Z.-H. (2011). Influence of curriculum reform: an analysis of student mathematics achievement in Mainland China. Int. J. Educ. Res. 50, 100–116. 10.1016/j.ijer.2011.06.005

[B40] Nishimura T. Seo M. Uesaka Y. Manalo E. Tanaka E. Ichikawa S.. (2017). Development of a scale about failure beliefs in academic activities. Japanese J. Educ. Psychol. 65, 197–210. 10.5926/jjep.65.197

[B41] Pajares F. (1996). Self-efficacy beliefs in academic settings. Rev. Educ. Res. 66, 543–578. 10.3102/00346543066004543

[B42] Pajares F. Miller M. D. (1994). Role of self-efficacy and self-concept beliefs in mathematical problem solving: a path analysis. J. Educ. Psychol. 86, 193–203. 10.1037/0022-0663.86.2.193

[B43] Passolunghi M. C. Ferreira T. I. R. Tomasetto C. (2014). Math–gender stereotypes and math-related beliefs in childhood and early adolescence. Learn. Individ. Dif. 34, 70–76. 10.1016/j.lindif.2014.05.005

[B44] Patterson M. M. Kravchenko N. Chen-Bouck L. Kelley J. (2016). General and domain-specific beliefs about intelligence, ability, and effort among preservice and practicing teachers. Teach. Teacher Educ. 59, 180–190. 10.1016/j.tate.2016.06.004

[B45] Rhew E. (2017). The effect of a growth mindset intervention on self-efficacy and motivation of adolescent special education students. Education Dissertations 6.

[B46] Rhew E. Piro J. S. Goolkasian P. Cosentino P. Palikara O. (2018). The effects of a growth mindset on self-efficacy and motivation. Cogent Educ. 5, 1–16. 10.1080/2331186X.2018.1492337

[B47] Robins R. W. Pals J. L. (2002). Implicit self-theories in the academic domain: implications for goal orientation, attributions, affect, and self-esteem change. Self Identity 1, 313–336. 10.1080/15298860290106805

[B48] Samuel T. S. Warner J. (2019). “I can math!”: reducing math anxiety and increasing math self-efficacy using a mindfulness and growth mindset-based intervention in first-year students. Commun. Coll. J. Res. Pract. 45, 1–8. 10.1080/10668926.2019.1666063

[B49] Schleicher A. (2019). PISA 2018: Insights and Interpretations. Available online at: https://www.oecd.org/pisa/PISA%202018%20Insights%20and%20Interpretations%20FINAL%20PDF.pdf (accessed January 12, 2019).

[B50] Schunk D. H. (1989). Self-efficacy and achievement behaviors. Educ. Psychol. Rev. 1, 173–208. 10.1007/BF01320134

[B51] Schunk D. H. Hanson A. R. (1985). Peer models: influence on children's self-efficacy and achievement. J. Educ. Psychol. 77, 313–322. 10.1037/0022-0663.77.3.313

[B52] Song J. Kim S. I. Bong M. (2020). Controllability attribution as a mediator in the effect of mindset on achievement goal adoption following failure. Front. Psychol. 10:2943. 10.3389/fpsyg.2019.02943 32010019 PMC6974511

[B53] Spinath B. Spinath F. M. Riemann R. Angleitner A. (2003). Implicit theories about personality and intelligence and their relationship to actual personality and intelligence. Pers. Individ. Dif. 35, 939–951. 10.1016/S0191-8869(02)00310-0

[B54] Stern M. Hertel S. (2020). Profiles of parents' beliefs about their child's intelligence and self-regulation: a latent profile analysis. Front. Psychol. 11:610262. 10.3389/fpsyg.2020.610262 33362670 PMC7756061

[B55] Stevenson H. W. Lee S.-Y. Chen C. Stigler J. W. Hsu C.-C. Kitamura S.. (1990). Contexts of achievement: a study of American, Chinese, and Japanese children. Monogr. Soc. Res. Child Dev. 55:123. 10.2307/1166090 2342493

[B56] Vandenberg R. J. Lance C. (2000). A review and synthesis of the measurement invariance literature: suggestions, practices, and recommendations for organizational research. Organ. Res. Methods 5, 139–158. 10.1177/109442810031002

[B57] Wang M. T. Degol J. L. (2017). Gender gap in science, technology, engineering, and mathematics (STEM): current knowledge, implications for practice, policy, and future directions. Educ. Psychol. Rev. 29, 119–140. 10.1007/s10648-015-9355-x 28458499 PMC5404748

[B58] Weiner B. (1986). An attributional theory of motivation and emotion. SSSP Springer 92, 159–190. 10.1007/978-1-4612-4948-1 3903815

[B59] Whittington R. E. Susan R. Daphne L. Ian H. (2017). Exploring the link between mindset and psychological well-being among veterinary students. J. Vet. Med. Educ. 44, 134–140. 10.3138/jvme.1215-192R 28206831

[B60] Yao J. Yang L. (2017). Perceived prejudice and the mental health of chinese ethnic minority college students: the chain mediating effect of ethnic identity and hope. Front. Psychol. 8:1167. 10.3389/fpsyg.2017.01167 28744249 PMC5504151

[B61] Zeng G. Hou H. Peng K. (2016). Effect of growth mindset on school engagement and psychological well-being of chinese primary and middle school students: the mediating role of resilience. Front. Psychol. 7:1873. 10.3389/fpsyg.2016.01873 28018251 PMC5147462

[B62] Zhang J. Kuusisto E. Tirri K. (2017). How teachers' and students' mindsets in learning have been studied: research findings on mindset and academic achievement. Psychology 8, 1363–1377. 10.4236/psych.2017.89089

[B63] Zhao Y. Niu G. Hou H. Zeng G. Xu L. Peng K.. (2018). From growth mindset to grit in chinese schools: the mediating roles of learning motivations. Front. Psychol. 9:2007. 10.3389/fpsyg.2018.02007 30405492 PMC6200841

